# Can we prevent or treat multiple sclerosis by individualised vitamin D supply?

**DOI:** 10.1186/1878-5085-4-4

**Published:** 2013-01-29

**Authors:** Jan Dörr, Andrea Döring, Friedemann Paul

**Affiliations:** 1 NeuroCure Clinical Research Center, Charité - Universitätsmedizin Berlin, Charitéplatz 1, Berlin, 10117, Germany; 2 Clinical and Experimental Research Center for Multiple Sclerosis, Charité - Universitätsmedizin Berlin, Berlin, Germany; 3 Current address: Department of Neurology, DIAKO, Flensburg, Germany

**Keywords:** Multiple sclerosis, Vitamin D, Cholecalciferol, Prevention, Therapy, Risk factor, Supplementation, Personalised medicine, Targeted prevention, Tailored therapy

## Abstract

Apart from its principal role in bone metabolism and calcium homeostasis, vitamin D has been attributed additional effects including an immunomodulatory, anti-inflammatory, and possibly even neuroprotective capacity which implicates a possible role of vitamin D in autoimmune diseases like multiple sclerosis (MS). Indeed, several lines of evidence including epidemiologic, preclinical, and clinical data suggest that reduced vitamin D levels and/or dysregulation of vitamin D homeostasis is a risk factor for the development of multiple sclerosis on the one hand, and that vitamin D serum levels are inversely associated with disease activity and progression on the other hand. However, these data are not undisputable, and many questions regarding the preventive and therapeutic capacity of vitamin D in multiple sclerosis remain to be answered. In particular, available clinical data derived from interventional trials using vitamin D supplementation as a therapeutic approach in MS are inconclusive and partly contradictory. In this review, we summarise and critically evaluate the existing data on the possible link between vitamin D and multiple sclerosis in light of the crucial question whether optimization of vitamin D status may impact the risk and/or the course of multiple sclerosis.

## Review

### Multiple sclerosis: background information

Multiple sclerosis (MS) is the most common chronic inflammatory disease of the central nervous system (CNS) in young adults in Western countries and often leads to early disability and retirement [1]. Typical clinical manifestations are optic neuritis, central paralysis, sensory disturbances, and difficulties in coordination and balance, as well as cognitive dysfunction, fatigue, and sleep disorders [1-3]. The initial course is usually relapsing-remitting, but after several years, the disease tends to convert into a secondary progressive form. A primary progressive course also exists but is much less common. It is estimated that 2.5 million people suffer from MS worldwide, and as in most autoimmune disorders, there is an obvious female preponderance of approximately 3 to 4:1 [1,4]. Importantly, most female patients are affected in their child-bearing age which may have fundamental consequences for family planning [5]. The cause of MS is not yet clear. Several genetic and environmental factors have been isolated to contribute to the risk of MS, among them vitamin D (VD) status, but the individual significance of each factor is not yet clear [6-10]. From the pathophysiological point of view, dysregulated encephalitogenic T cells are thought to initiate and to orchestrate in concert with abundant other immune cells an autoimmune multifocal CNS inflammation [11-13]. For decades, MS was considered to be a primarily demyelinating disorder predominantly affecting the CNS white matter. During recent years, however, it has become clear that MS also has a strong neurodegenerative component, which is most probably the underlying basis for the development of permanent disability [14-17]. Moreover, grey matter involvement has been increasingly recognised by means of histopathology and magnetic resonance imaging (MRI) [18-20]. Paraclinical tools for diagnosis, differential diagnosis, and monitoring of disease activity and progression in MS comprise cerebrospinal fluid examination, evoked potentials, MRI, and recently, optical coherence tomography [1,21-24].

Current treatment concepts comprise the application of immunomodulatory or immunosuppressive drugs such as interferon-beta, glatiramer acetate, fingolimod, natalizumab, or mitoxantrone [25,26]. Approval of additional new drugs is expected. However, most if not all of these drugs lack convincing neuroprotective capacity. Moreover, a substantial number of patients do not respond satisfyingly to these drugs or experience severe side effects [27-31]. Overall, there is still a need for improved therapeutic approaches, especially in neuroprotective substances [32].

### Vitamin D: background information

Research on VD started around 1915, stimulated by the quest for an effective treatment of rickets. By the end of the 19th century, up to 90% of the children living in large cities throughout Northern Europe and the United States suffered from rickets, and the most common cause was the insufficient supply of VD due to low sun exposure as a side effect of increasing industrialisation. The transformation to an industrialised economy radically changed the living conditions for large parts of the population. Children often had to work many hours a day in factories or mines, being completely shielded from the sun. When VD deficiency was recognised as the main cause of rickets, a significant reduction of cases was achieved by preventive measures like radiation from ultraviolet lamps, greater amount of time spent outdoors, or fortification of food with VD [33].

The VD supply of the human organism is generally accomplished via two different routes: first, endogenous synthesis of VD3 (cholecalciferol) from its precursor 7-dehydrocholesterol in an ultraviolet (UV) B radiation-dependent process in the skin (wave length 290 to 315 nm); second, exogenous supply with VD3 or VD2 (ergocalciferol) by food, fortified food products, or supplements [34,35]. About 90% to 100% of the VD requirement of a human body is covered by sun exposure-dependent endogenous production [33,36]. The amount of UVB-radiation dependent VD production depends on numerous factors including individual factors like duration and frequency of sun exposure, the area of skin exposed to the sun, use of sun protection, skin pigmentation, age, sex, genetic factors, amounts of 7-dehydrocholesterol in the skin; geographic factors like latitude and altitude; as well as seasonal and meteorological factors like clouding and ozone levels [36]. The magnitude of endogenous VD synthesis is referenced to the minimum erythema dose (MED) which describes the minimum individual dose of UVB radiation needed for the development of a transient skin irritation. One MED of the entire body equals the release of 10,000 to 20,000 IU (250 to 500 μg) of VD3 [1]. Compared to the endogenous production of VD3, the food-related intake of VD2/3 is usually of inferior importance since only few food products contain significant amounts of VD (Table 1).

**Table 1:** **Dietary sources of vitamin D**[37]

**Food product**	**Vitamin D content (μg/100g)**	**Required daily intake (in g) for 20 μg vitamin D**
Cod liver oil	330	6
Smoked eel	22	91
Salmon	3.8	526
Avocado	3.43	583
Egg yolk	2.9	690
Liver (beef)	1.7	1,176
Butter	1.2	1,667
Pork	0.11	18,182
Milk (3.5%)	0.088	22,727

Both VD2 and VD3 are biologically inactive. After intradermal synthesis or intestinal uptake, VD2 and VD3 are bound mainly to vitamin-D-binding-protein and transported to the liver, where they are enzymatically hydroxylated to 25(OH)VD (calcidiol). As this step is not tightly regulated and because of the relatively long half-life, serum levels of 25(OH)VD integrate both the endogenous and exogenous supply and provide a good estimate of an organism's VD status. The enzyme 1α-hydroxylase (CYP27B1), which is located mainly in the kidneys but also in other tissues, converts 25(OH)VD in a second hydroxylation step into the biologically active 1,25 dihydroxyvitamin D (1,25(OH)_2_VD; calcitriol) [35]. Unlike the first hydroxylation, this second step is tightly regulated, among others by parathormone and calcium/phosphate levels [38]. Calcitriol effects are mainly mediated via the intracellular VD receptor (VDR) which functions as a transcription factor and controls the expression of numerous genes. In its membrane-bound form, VDR mediates additional non-genomic functions including several signal transduction pathways [39,40].

An ongoing debate addresses the optimal serum levels of 25(OH)VD. Currently, most experts consider 25(OH)VD levels above 30 ng/ml (75 nmol/l) as adequate [41-43], which is supported by the observations that serum levels of parathormone start plateauing at serum 25(OH)VD of 30 to 40 ng/ml and that immunological effects need serum levels around 30 ng/ml [35,44]. Levels below 20 ng/ml (50 nmol/l) are considered deficient. Less clear are the upper limits since substantial variability occurs in naturally occurring 25(OH)VD levels. According to the literature, levels of up to 150 to 200 ng/ml (375 to 500 nmol/l) can be considered safe [41]. Against this background, a significant proportion of the human population worldwide shows an alarming VD inadequacy [35,44-46].

Since VD homeostasis is linked on multiple levels to the risk of not only various diseases such as cancer and autoimmune diseases, but also metabolic, cardiovascular, and psychiatric disorders [35,42,47,48], the question arises whether improvement of VD supply may prevent or even treat respective diseases. Indeed, recent estimations indicate that yearly, >110,000 deaths could be prevented by adequate VD supply [49].

### Linking vitamin D and MS: immunoregulatory functions of vitamin D

**Figure 1:**
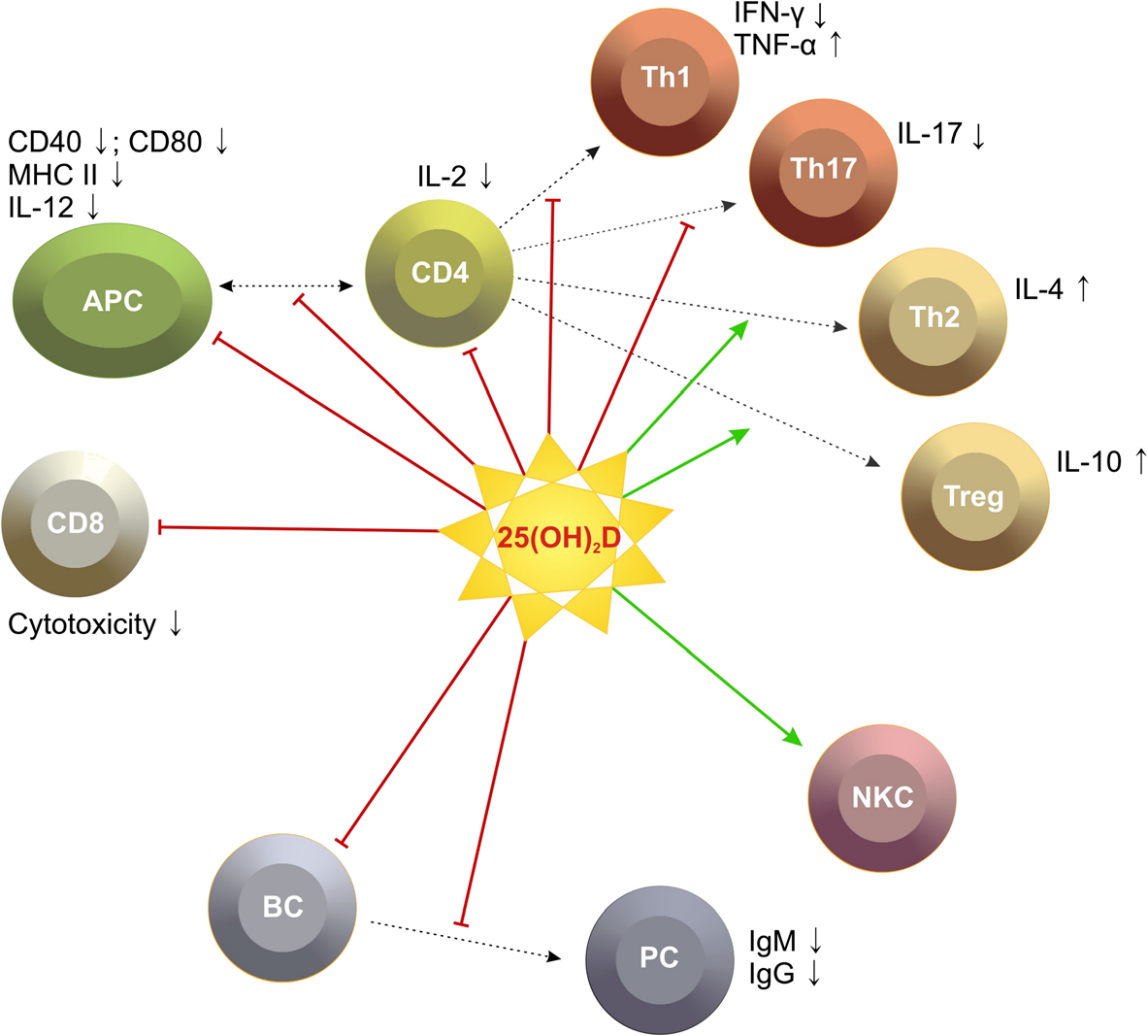
**Possible effects of vitamin D on immune cells.** APC, antigen presenting cell; Th, T helper cell; Treg, regulatory T cell; BC, B cell; PC, plasma cell; NKC, natural killer cell. Figure was first published in [62] .

Apart from its fundamental role in calcium homeostasis and bone metabolism, increasing evidence suggests that VD has additional, particularly immunoregulatory functions which renders VD a promising candidate in both pathogenesis and treatment of autoimmune diseases such as MS. The capability of VD to modulate both innate and adaptive immune responses has been summarised in several comprehensive and excellent reviews [47,50-52]. With respect to the autoimmune MS pathophysiology [11,12], the following effects of VD on the immune system might be of particular interest: the ability to modulate the differentiation and function of antigen presenting cells which results in a reduced activation of potentially auto-aggressive T cells [53-55], the capacity to inhibit B cell and T cell proliferation and differentiation [56-58], the ability to shift the cytokine milieu from a pro-inflammatory, Th1/Th17-cell-mediated to a rather anti-inflammatory Th2-cell-mediated state [47,59], and finally, to facilitate the differentiation of regulatory T cells and function of natural killer cells [60,61]. Data on the VD effect on CD8 cells are still controversial. Figure 1 summarises the potential immunoregulatory effects of VD that might be pathophysiologically relevant in MS.

The presence of 1α-hydroxylase activity in neurons and microglia, and the presence of VD receptor in the CNS suggest local-, paracrine-, or autocrine-mediated effects of VD in the CNS [63,64]. Interestingly, data from *in vitro* or animal studies suggest that neurotrophic factors such as nerve growth factor, neurotrophin 3, and glial cell line-derived neurotrophic factor are regulated by VD which might indicate additional, possibly neuroprotective effects of VD [65]. Whether VD has clinically relevant neuroprotective properties still remains a subject of discussion.

### Linking vitamin D and MS: how do genes contribute?

It is long known that genetic factors contribute to the risk of MS. In particular, an association with extended major histocompatibility complex haplotypes, especially those containing HLA-DRB1*1501, has been consistently shown in individuals of northern European ancestry [66,67]. The role of VD-related genes in determining MS risk or specific genetic interactions with VD is currently a hot focus of research and is not yet completely understood. So far, two interesting links merit mentioning: First, it was recently shown that the gene expression of allele HLA-DRB1*1501 is modulated by VD, and a highly conserved VD-responsive element has been identified in the promoter region of the HLA-DRB1*1501 haplotype, which may indicate a direct functional interaction between VD and the major locus determining genetic susceptibility to MS [68]. Second, loss of function variants in the *CYP27B1* gene which encodes the enzyme that converts 25(OH)VD into its active form were shown to be associated with an increased MS risk [69]. In the same direction points a possible association between MS and VD-dependent rickets type I, which is a rare hereditary condition caused by a mutation in *CYP27B1*[70,71].

### Linking vitamin D and MS: what do animal models tell us?

Further evidence for a causal relation between VD supply and both development and treatment of MS were derived from experimental autoimmune encephalomyelitis (EAE), the best established rodent animal model for MS. In murine EAE, prophylactic application of VD (starting at disease induction) resulted in a reduction of both disease incidence and severity. Likewise, the therapeutic VD application (starting at onset of symptoms) lead to a significant reduction of disease severity [72-74]. Interestingly, some studies suggested gender-specific efficacy of VD only in female mice [75]. In a recent study, continuous treatment of mice with UVR dramatically suppressed clinical signs of EAE. Interestingly, the therapeutic effect was paralleled by only a moderate and transient increase of serum 25(OH)VD levels, which suggests that directly UVR-mediated effects which were at least partly independent of VD contributed to this observation [76]. In another recent study using the cuprizone model, dietary VD could (partly) prevent chemically induced CNS demyelination in mice [77].

### Linking vitamin D and MS: the clue to geographic and seasonal associations?

First hypotheses on a possible link between MS risk and VD deficiency were derived from the observation that the risk of MS is associated with latitude [78,79] which in turn shows a strong inverse correlation with UVB exposure. Furthermore, migrating from high to low latitude appears to reduce the MS risk [80]. This link was further corroborated by the observation of a MS risk lower than one would expect from the latitude in regions with a high consume of fatty VD-rich fish [81]. More recent investigations, however, suggest that this latitude gradient is fading which might be explained by several possible reasons, including better MS recognition, changes in lifestyle, and improvement of sanitary circumstances [34]. More indirect though not unambiguous support for a beneficial effect of VD comes from the observation that both MS risk and disease activity show a seasonal association. As shown in several studies including a very recent meta-analysis, humans born in spring have a significant higher risk to develop MS later in life than people born in autumn [82-85] which might be at least partially explained by longer in utero VD insufficiency due to lower motherly VD levels in winter/spring as compared to summer/autumn. Likewise, several methodically high quality studies showed an inverse association between sun exposure or outdoor activities during childhood and adolescence, and the risk of developing MS during adulthood [86-90]. In line with these reports is the recent observation that low sun exposure in fall/winter before disease onset was associated with a less favourable outcome [91]. Yet, all these studies have two major intrinsic limitations: first, despite a reported reasonable validity and reliability [92], the retrospective determination of sun exposure years or even decades in the past is inevitably subjected to recall bias [34], and prospective studies are hardly available. The determination of actinic damage as a surrogate parameter for cumulative sun exposition might be a viable loophole [34,86]. Second, sun exposure itself may have intrinsic immunomodulatory effects, independent of VD [76,93,94]. Also, not easy to harmonise with sun exposure or VD synthesis is the seasonal dependency of disease activity in already established MS. Several studies including a meta-analysis showed an excess of clinical exacerbations and MRI activity in spring/summer and a nadir in autumn/winter in the northern hemisphere [95-98]. Correspondingly, a reverse situation was observed in the southern hemisphere [99]. While a peak of disease activity in spring and a nadir in autumn in the northern hemisphere could be explained with a few-month lag in the course of serum VD levels, the situation in summer and winter does not easily fit with a protective role of VD. In conclusion, VD might contribute to some but cannot sufficiently explain all geographic and seasonal associations observed in MS.

### Linking vitamin D and MS: the impact of vitamin D intake and serum level

The rather indirect impact of predictors of 25(OH)VD levels on MS has been discussed above. But, how does the 25(OH)VD serum level itself sway the risk and course of MS? Generally, if VD had a beneficial effect on MS risk, one would demand an inverse relation between VD intake or serum levels and MS incidence. Indeed, various studies demonstrated such a relation. Most data on this issue, however, are derived from epidemiologic or observational studies, meaning, that methodical limitations like selection bias, retrospective survey, and interference with various confounders should be kept in mind. One recent study suggests that already in utero levels of VD, which are completely dependent on the mother's VD status, impact the risk to develop MS later in life [100]. In a Canadian cohort study on children presenting with a first demyelinating event, the risk to develop definite MS within the following 3 years was inversely and independently correlated with the 25(OH)VD serum level [101]. Furthermore, data from a nested case–control study involving more than seven million individuals of the US military suggest that in healthy young white adults, higher 25(OH)VD levels are predictive of a significantly lower risk of developing MS (62% lower odds in the top quintile of 25(OH)VD serum levels compared to the bottom quintile), independent from latitude of residency in childhood [102]. Another study by the same group addressed the relation between VD intake and MS risk in a cohort of approximately 200,000 US women and reported a 33% reduction of MS incidence over a follow-up period of 30 years when comparing the top quintile and the bottom quintile of VD intake. Moreover, in women taking daily supplements containing at least 400 IU VD, a 41% lower MS incidence was observed when compared to women who did not take supplements [103]. Likewise, in another survey, intake of cod liver oil was associated with a 4-year delay of MS onset [90]. In summary, substantial evidence exists for an inverse association between VD and the risk of developing MS.

But, how does the situation look in already established MS? A number of studies consistently suggest that higher VD serum levels are associated with a more favourable disease course. In a small Finnish study, lower summer 25(OH)VD concentrations were measured in patients with a first MS relapse compared to healthy controls, and 25(OH)VD concentrations were significantly lower during relapses than in remission phases which may point to a regulative role of VD for MS activity [104]. Compelling support for this hypothesis comes from four independent recent reports, all showing a close relationship between clinical disease activity and 25(OH)VD concentrations: Two studies demonstrated that every 10 nmol/l increase of the VD serum level is correlated with a reduction of relapse occurrence of 11% and 13.7%, respectively [105,106]. A third study demonstrated a log linear association between serum VD concentration and MS relapse rate in that every doubling of serum levels reduced relapse rate by 27% [107]. The fourth study finally revealed a 34% reduction of relapse rate by every 10 ng/ml increase in paediatric onset MS [108]. In line with these clinical data, an inverse association between VD concentrations and disease activity on cranial MRI was recently demonstrated, but may possibly be restricted to patients without additional immunomodulatory treatment [109,110]. Of note, in studies addressing the relation between clinical disease activity and VD levels, a reverse association (low VD concentrations as a consequence rather than a cause of a relapse) cannot be completely ruled out. In contrast to the serum concentrations, a statistical difference in cerebrospinal fluid VD levels was neither observed between MS patients and healthy controls or in MS patients between phases of disease activity or in remission [111].

In conclusion, cumulating evidence quite consistently argues for a relationship between VD status and both risk and activity of MS. Of note, however, all these studies are methodically prone to bias and are therefore not suited to definitely proof such a relation.

### Linking vitamin D and MS: what do interventional trials tell us?

The compelling evidence for the beneficial impact of higher VD serum concentrations on disease activity leads directly to two questions: (a) do patients with already established MS benefit from a therapeutic elevation of their VD levels and (b) if so, which 25(OH)VD serum levels should be strived for in MS patients? To reliably answer these crucial questions, high quality and sufficiently powered interventional trials are required. Moreover, important issues like optimal dosing schemes need further clarification. So far, there are only few prospective clinical studies on VD as a treatment for MS, some of them of rather questionable quality. An early uncontrolled study involving 16 MS patients (evidence level IIb) showed that regular intake of cod liver oil (equivalent to 5,000 IU VD/day) for a period of up to 2 years lead to a lower relapse rate as would have been expected from the participants' medical histories [112]. From today's point of view, design and sample size of this study are however not appropriate to address a therapeutic effect of VD. Another small and uncontrolled study with a primary focus on safety aspects (evidence level IIb) provided evidence that escalating VD doses up to 280,000 IU/week over a rather short period of 28 weeks are safe in MS patients. No significant effects on clinical parameters were observed, but there was a possible effect on MRI activity [113]. In a successive randomised controlled but open label study, the same group applied cholecalciferol (up to 40,000 or 4,000 IU/d) continuously for 52 weeks in 49 MS patients (evidence level Ib). Patients in the high dose arm showed a significant reduction of the annualised relapse rate [114]. In another randomised double blind and placebo-controlled study focusing on serological markers of disease activity (evidence level IIb), administration of 1,000 IE cholecalciferol for a period of 6 months lead to a significant increase of the anti-inflammatory cytokine transforming growth factor-β and to a partial reduction of the IL-2 level [115]. Another recent study (evidence level IIb) reported no significant differences between the high- or low-dose ergocalciferol on clinical and MRI parameters [116]. However, the design of this study (small number, inhomogeneous group of patients, short period of observation, relatively high dosages of “low-dose” ergocalciferol) has been criticised as not being suited to address the therapeutic capacity of VD in MS [117]. The capacity of low dose calcitriol to prevent disease progression in relapsing remitting multiple sclerosis (RRMS) when given in adjunction to undefined disease-modifying treatment strategies was investigated in a randomised and double blind but rather small study on 50 patients. After 12 months, no significant differences between verum and placebo arms were observed with respect to relapse rate and disability [118]. Two recently published studies from Finland and Norway, both applying 20,000 IU/week in a randomised, double blind and placebo-controlled design (evidence level Ib), yielded partly contradictory results with respect to clinical and MRI parameters. In the Finish study, mean 25(OH)VD serum levels in patients receiving VD over 1 year in addition to IFN-β increased to 110 nmol/l, and patients in the verum group showed significantly fewer gadolinium-enhancing lesions and a tendency to reduced disability accumulation and improved ambulation parameters. The annualised relapse rate was not different in both arms [119]. In an active subgroup of this study, an additional beneficial effect of VD on new/enlarging T2 hyperintense brain lesions was observed [120]. In the 96-week Norwegian trial, no significant differences were observed in annualised relapse rate, EDSS, MSFC, grip strength, or fatigue although the median 25(OH)VD serum concentration in the verum group raised to 121 nmol/l [121].

In summary, due to their ambiguous results, the so-far published interventional trials do not answer the question whether VD would be a treatment option in MS. The reasons for these heterogeneous results remain unclear. Given the substantial increase in serum concentration to greater 100 nmol/l in the two most recent trials [119,121], insufficient dosing is probably not a likely explanation. Further, well-designed interventional high-dose trials, which are at least partly better powered, are currently underway (Table 2) [122,123] and will hopefully contribute to elucidate the efficacy aspects.

**Table 2:** **Ongoing clinical trials on vitamin D in multiple sclerosis (registered at http://www.clinicaltrials.gov by January 2013)**

**Trial title/registration number**	**Sponsor**	**Start date/estimated completion date**	**Intervention**	**Trial design**	**Number of participants**	**Main outcome parameters**
Phase II study of efficacy of vitamin D supplementation in multiple sclerosis (EVIDIMS study)	Charité-Universitätsmedizin Berlin, Germany	December 2011/March 2015	Cholecalciferol 20,400 IU every other day (high dose) or cholecalciferol 400 IU/every other day (low dose) for 18 months	Randomised double blind active controlled multicenter Phase II trial	80 patients with RRMS or CIS	Primary: cumulative number of new T2 lesions
NCT01440062			Add-on to IFN β1b 250 μg every other day			Secondary: annualised relapse rate, occurrence of disability progression, proportion of patients without disease activity, conversion rate into definite MS, cumulative number of T1 gadolinium-enhancing lesion, number and volume of new T1 hypointense lesions, number and volume of new T2 hyperintense lesions, changes in brain parenchymal volume, changes in magnet resonance spectroscopy, changes in retinal structure as determined by optical coherence tomography, changes in cognitive function and fatigue, change in health-related quality of life
Supplementation of VigantOL® oil versus placebo as add-on in patients with relapsing remitting multiple sclerosis receiving Rebif® treatment (SOLAR study)	Merck-Serono GmbH	February 2011/ March 2014	Cholecalciferol 14,000 IU/day or placebo for 96 weeks	Randomised double blind placebo-controlled multicenter phase II trial	348 patients with RRMS	Primary: mean number of active lesions at week, proportion of relapse-free subjects
NCT01285401			Add-on to IFN β1a 44 μg 3×/week			Secondary: annualised relapse rate, proportion of subjects free from any EDSS progression, proportion of subjects free from disease activity, change in cognitive function, cumulative number of T1 gadolinium enhancing lesion, proportion of subjects free from new T1 hypointense lesions, change from baseline in the total volume of T2 lesions, percent brain volume change with respect to baseline
A multicentre, randomised, double-blind, placebo-controlled study of the efficacy of supplementary treatment with cholecalciferol in patients with relapsing multiple sclerosis treated with subcutaneous IFN Beta-1a 44 μg 3 times weekly	Merck KGaA	January 2010/July 2014	Cholecalciferol 2× 100,000 IU/month or placebo for 96 weeks	Randomised double blind placebo-controlled multicenter phase II trial	250 patients with RRMS	Primary: reduction of relapse rate
NCT01198132			Add-on to IFN β1a 44 μg 3×/week			Secondary: number of relapse-free subjects, cumulative probability of progression of disability, number of new or extended lesions in T1- and T2-weighted MRI, changes in lesion load (T2), number of new lesions (T1 gadolinium activity and black holes), measurement and evaluation of cognitive ability, change in quality of life, safety of the treatment
A pilot study to assess the relative safety and immunology effects of low dose versus high dose cholecalciferol supplementation in patients with multiple sclerosis	Johns Hopkins University, Baltimore, USA	March 2010/December 2011	Cholecalciferol 10,000 IU/day (high dose) or cholecalciferol 400 IU/day (low dose) for 6 months	Randomised double blind controlled multicenter phase II trial	40 MS patients with or without immunomodulatory treatment and serum 25(OH)VD levels between 20–50 ng/ml	Primary: safety of high-dose cholecalciferol, effects of cholecalciferol supplementation on serum immune markers
NCT01024777						Secondary: clinical effects of cholecalciferol supplementation
A randomised controlled trial of vitamin D supplementation in multiple sclerosis	Johns Hopkins University, Baltimore, USA	March 2012/December 2014	Cholecalciferol 5,000 IU/day (high dose) or cholecalciferol 600 IU/day (low dose) for 24 months	Randomised double blind controlled multicenter phase III trial	172 RRMS patients with 25(OH)D-serum levels ≥ 15 ng/ml	Primary: proportion of subjects that experience a relapse
NCT01490502			Add-on to glatiramer acetate 20 mg/day			Secondary: annualised relapse rate, occurrence of sustained disability progression, number of new T2 lesions, changes in brain parenchymal volume and cortical thickness, change in low-contrast visual acuity, change in health-related quality of life, development of hypercalcemia/related adverse effects
Pharmacodynamic and immunologic effects of vitamin D supplementation in patients with multiple sclerosis and healthy controls	Johns Hopkins University, Baltimore, USA	November 2010/June 2013	Cholecalciferol 5,000 IU/day for 90 days	Non-randomised, open label single group assignment multicenter phase 1 trial	60 patients with RRMS or healthy individuals	Primary: change in mean serum level of 25(OH)VD
NCT01667796						Secondary: cytokine levels and percentages of T and B cells, gene expression microarray
Role of vitamin D on the relapse rate of multiple sclerosis	AlJohara M AlQuaiz, M.D., King Saud University, Saudi Arabia	January 2013/October 2014	Cholecalciferol 50,000 IU/week or placebo for 12 months	Randomised double blind controlled single centre phase II trial	200 patients with RRMS	Primary: relapse rate
NCT01753375						Secondary: improvement in the EDSS score
Dose-related effects of vitamin D3 on immune responses in patients with clinically isolated syndrome and healthy control participants. An exploratory double blind placebo randomised controlled study	University College Dublin, Ireland	November 2012/May 2014	Cholecalciferol 5,000 IU/day or 10,000 IU/day or placebo for 24 weeks	Randomised double blind placebo-controlled phase II trial	45 patients with CIS without any immunomodulatory treatment and 39 healthy individuals	Primary: change in the frequency of CD4 T cell subsets and cytokine responses of periphery blood mononuclear cells
NCT01728922						Secondary: relapse occurrence, percentage of CIS patients in each treatment arm free from any evidence of disease activity, number of new T2 and gadolinium-enhancing lesions

Abbreviations: *CIS* clinically isolated syndrome, *EDSS* expanded disability status scale, *IFN* interferon, *IU* international units; *RRMS* relapsing remitting multiple sclerosis.

With respect to safety, more clinical data already exist. Generally, (iatrogenic) VD excess can result in life-threatening hypercalcaemia and has been occasionally reported on the basis of single cases [124]. However, unlike supplementation with high dose calcitriol, which indeed seems to bear a significant risk of symptomatic hypercalcaemia [125], treatment of MS patients with even very high doses of cholecalciferol or ergocalciferol was repeatedly demonstrated to be safe [113,114,116,119,121]. While a Cochrane report published in 2010 concludes that available data are not yet sufficient to draw the right conclusions regarding safety of VD supplementation [126], another recent meta-analysis suggests that daily doses of 10,000 IE cholecalciferol can be considered safe [127].

## Conclusions

In this review article, which follows the recommendations of the “EPMA White Paper” [128], we summarise and discuss available data on the role of VD for the development and disease course of MS. Many lines of evidence, in particular epidemiologic data, preclinical investigations, animal studies, and association studies on VD status and disease activity, suggest that higher serum concentrations of VD are beneficial in terms of the risk to develop MS as well as the further course of the disease in already-established MS. Moreover, VD supplementation is safe, cheap, and convenient to perform. Therefore, it is intriguing to hypothesise that boosting the VD serum levels would be an option to both prevent and treat MS. Despite the inherent methodological drawbacks of epidemiologic studies, existing data on the preventive capacity of higher VD levels are quite compelling. Final proof of this hypothesis would be reached by large-scale prospective epidemiological studies which will probably not be available in the near future, for obvious reasons. With respect to the therapeutic efficacy, an association between higher VD serum concentrations and a favourable disease course has been conclusively shown. Unfortunately, the so-far performed interventional trials, though not negotiating this hypothesis, also do not unambiguously support the idea that raising patients' VD levels would be favourably in terms of disease outcome. Hopefully, ongoing (Table 2) and future trials will shed more light on this aspect.

But, how are we going to deal with this issue in the meantime? From a pragmatical point of view and considering available data on efficacy, safety, tolerability, and last but not least costs, it seems to be reasonable to regularly control 25(OH)VD levels in MS patients, especially during winter months. In patients with inadequate VD, levels should be raised to at least 30–40 ng/ml (75–100 mmol/l), either by appropriate sun exposure and/or adequate VD supplementation. As a rule of thumb, supplementary 1 μg (40 IU) cholecalciferol will increase 25(OH)VD levels by 1 ng/ml (2.5 nmol/l).

## Competing interests

JD received a limited grant for clinical research on vitamin D by Bayer Healthcare, speaker honoraria by Bayer Healthcare, Novartis, and Teva, consultancy honoraria by Bayer Healthcare, and travel support by Merck-Serono, Novartis, and Bayer Healthcare. FP received speaker honoraria and travel support by Bayer Healthcare, Teva, Sanofi-Aventis/Genzyme, Biogen Idec, Novartis, and Merck-Serono. FP is supported by the Artur-Arnstein Foundation Berlin, Germany, and has received travel reimbursement from the Guthy Jackson Charitable Foundation. AD declares no competing interests.

## Authors’ contributions

AD and JD drafted the manuscript and did the literature search. FP critically revised the manuscript for important intellectual content. All authors read and approved the final manuscript.

## Authors’ information

JD is a neurologist and a senior physician/scientist at the NeuroCure Clinical Research Center (NCRC), Charité-Universitätsmedizin Berlin, Germany, and has a research focus on the role of VD in MS. As head of the NCRC Neuroimmunological Study Outpatient Department, he is currently running a therapeutic phase II clinical trial on VD in MS. AD is a physician; she completed part of her neurological training at the NCRC. FP is a senior neurologist and head of the Neuroimmunological Group at the NCRC. His research focus is the translation of new diagnostic tools and therapeutic approaches in MS and related disorders into the clinical setting.

## References

[B1] Compston A Coles A Multiple sclerosis Lancet 2008 372 1502 1517 10.1016/S0140-6736(08)61620-7 18970977

[B2] Weinges-Evers N Brandt AU Bock M Pfueller CF Dörr J Bellmann-Strobl J Scherer P Urbanek C Boers C Ohlraun S Zipp F Paul F Correlation of self-assessed fatigue and alertness in multiple sclerosis Mult Scler 2010 16 1134 1140 10.1177/1352458510374202 20610494

[B3] Veauthier C Radbruch H Gaede G Pfueller C Dörr J Bellmann-Strobl J Wernecke K-D Zipp F Paul F Sieb J Fatigue in multiple sclerosis is closely related to sleep disorders: a polysomnographic cross-sectional study Mult Scler 2011 17 613 622 10.1177/1352458510393772 21278050

[B4] Handel AE Jarvis L McLaughlin R Fries A Ebers GC Ramagopalan SV The epidemiology of multiple sclerosis in Scotland: inferences from hospital admissions PLoS One 2011 6 e14606 10.1371/journal.pone.0014606 21298053 PMC3029296

[B5] Borisow N Döring A Pfueller CF Paul F Dörr J Hellwig K Expert recommendations to personalization of medical approaches in treatment of multiple sclerosis: an overview of family planning and pregnancy EPMA J 2012 3 9 10.1186/1878-5085-3-9 22738272 PMC3464716

[B6] Ascherio A Munger KL Environmental risk factors for multiple sclerosis. Part I: the role of infection Ann Neurol 2007 61 288 299 10.1002/ana.21117 17444504

[B7] Ascherio A Munger KL Environmental risk factors for multiple sclerosis. Part II: noninfectious factors Ann Neurol 2007 61 504 513 10.1002/ana.21141 17492755

[B8] Handel AE Giovannoni G Ebers GC Ramagopalan SV Environmental factors and their timing in adult-onset multiple sclerosis Nat Rev Neurol 2010 6 156 166 10.1038/nrneurol.2010.1 20157307

[B9] Goodin DS The causal cascade to multiple sclerosis: a model for MS pathogenesis PLoS One 2009 4 e4565 10.1371/journal.pone.0004565 19242548 PMC2644781

[B10] Ramagopalan SV Dobson R Meier UC Giovannoni G Multiple sclerosis: risk factors, prodromes, and potential causal pathways Lancet Neurol 2010 9 727 739 10.1016/S1474-4422(10)70094-6 20610348

[B11] McFarland HF Martin R Multiple sclerosis: a complicated picture of autoimmunity Nat Immunol 2007 8 913 919 10.1038/ni1507 17712344

[B12] Nylander A Hafler DA Multiple sclerosis J Clin Invest 2012 122 1180 1188 10.1172/JCI58649 22466660 PMC3314452

[B13] Haegert DG Multiple sclerosis: a disorder of altered T-cell homeostasis Mult Scler Int 2011 2011 461304 22096637 10.1155/2011/461304 PMC3197186

[B14] Trapp BD Peterson J Ransohoff RM Rudick RA Mork S Bo L Axonal transection in the lesions of multiple sclerosis N Engl J Med 1998 338 278 285 10.1056/NEJM199801293380502 9445407

[B15] Peterson JW Bo L Mork S Chang A Trapp BD Transected neurites, apoptotic neurons, and reduced inflammation in cortical multiple sclerosis lesions Ann Neurol 2001 50 389 400 10.1002/ana.1123 11558796

[B16] De Stefano N Matthews PM Fu L Narayanan S Stanley J Francis GS Antel JP Arnold DL Axonal damage correlates with disability in patients with relapsing-remitting multiple sclerosis. Results of a longitudinal magnetic resonance spectroscopy study Brain 1998 121 Pt 8 1469 1477 9712009 10.1093/brain/121.8.1469

[B17] Vogt J Paul F Aktas O Muller-Wielsch K Dörr J Dörr S Bharathi BS Glumm R Schmitz C Steinbusch H Raine CS Tsokos M Nitsch R Zipp F Lower motor neuron loss in multiple sclerosis and experimental autoimmune encephalomyelitis Ann Neurol 2009 66 310 322 10.1002/ana.21719 19798635

[B18] Kutzelnigg A Lucchinetti CF Stadelmann C Brück W Rauschka H Bergmann M Schmidbauer M Parisi JE Lassmann H Cortical demyelination and diffuse white matter injury in multiple sclerosis Brain 2005 128 2705 2712 10.1093/brain/awh641 16230320

[B19] Sinnecker T Mittelstaedt P Dörr J Pfueller CF Harms L Niendorf T Paul F Wuerfel J Multiple sclerosis lesions and irreversible brain tissue damage: a comparative ultrahigh-field strength magnetic resonance imaging study Arch Neurol 2012 69 739 745 22351849 10.1001/archneurol.2011.2450

[B20] Sinnecker T Dörr J Pfueller CF Harms L Ruprecht K Jarius S Brück W Niendorf T Wuerfel J Friedemann P Distinct lesion morphology at 7-T MRI differentiates neuromyelitis optica from multiple sclerosis Neurology 2012 79 708 714 10.1212/WNL.0b013e3182648bc8 22855861

[B21] Bock M Brandt AU Dörr J Kraft H Weinges-Evers N Gaede G Pfueller CF Herges K Radbruch H Ohlraun S Bellmann-Strobl J Kuchenbecker J Zipp F Paul F Patterns of retinal nerve fiber layer loss in multiple sclerosis patients with or without optic neuritis and glaucoma patients Clin Neurol Neurosurg 2010 112 647 652 10.1016/j.clineuro.2010.04.014 20452719

[B22] Oberwahrenbrock T Schippling S Ringelstein M Kaufhold F Zimmermann H Keser N Young KL Harmel J Hartung H-P Martin R Paul F Aktas O Brandt AU Retinal damage in multiple sclerosis disease subtypes measured by high-resolution optical coherence tomography Mult Scler Int 2012 2012 530305 22888431 10.1155/2012/530305 PMC3410317

[B23] Dörr J Wernecke KD Bock M Gaede G Wuerfel JT Pfueller CF Bellmann-Strobl J Freing A Brandt AU Friedemann P Association of retinal and macular damage with brain atrophy in multiple sclerosis PLoS One 2011 6 e18132 10.1371/journal.pone.0018132 21494659 PMC3072966

[B24] Polman CH Reingold SC Banwell B Clanet M Cohen JA Filippi M Fujihara K Havrdova E Hutchinson M Kappos L Lublin FD Montalban X O'Connor P Sandberg-Wollheim M Thompson AJ Waubant E Weinshenker B Wolinsky JS Diagnostic criteria for multiple sclerosis: 2010 revisions to the McDonald criteria Ann Neurol 2011 69 292 302 10.1002/ana.22366 21387374 PMC3084507

[B25] Río J Comabella M Montalban X Multiple sclerosis: current treatment algorithms Curr Opin Neurol 2011 24 230 237 10.1097/WCO.0b013e328346bf66 21499098

[B26] Buck D Hemmer B Treatment of multiple sclerosis: current concepts and future perspectives J Neurol 2011 258 1747 1762 10.1007/s00415-011-6101-2 21637950

[B27] Jain N Bhatti MT Fingolimod-associated macular edema: incidence, detection, and management Neurology 2012 78 672 680 10.1212/WNL.0b013e318248deea 22371414

[B28] Paul F Dörr J Wurfel J Vogel HP Zipp F Early mitoxantrone-induced cardiotoxicity in secondary progressive multiple sclerosis J Neurol Neurosurg Psychiatry 2007 78 198 200 10.1136/jnnp.2006.091033 17229751 PMC2077678

[B29] Sorensen PS Bertolotto A Edan G Giovannoni G Gold R Havrdova E Kappos L Kieseier BC Montalban X Olsson T Risk stratification for progressive multifocal leukoencephalopathy in patients treated with natalizumab Mult Scler 2012 18 143 152 10.1177/1352458511435105 22312009

[B30] Giovannoni G Southam E Waubant E Systematic review of disease-modifying therapies to assess unmet needs in multiple sclerosis: tolerability and adherence Mult Scler 2012 18 932 946 10.1177/1352458511433302 22249762

[B31] Limmroth V Putzki N Kachuck NJ The interferon beta therapies for treatment of relapsing-remitting multiple sclerosis: are they equally efficacious? A comparative review of open-label studies evaluating the efficacy, safety, or dosing of different interferon beta formulations alone or in combination Ther Adv Neurol Disord 2011 4 281 296 10.1177/1756285611413825 22010041 PMC3187676

[B32] Fox RJ Primary neuroprotection: the Holy Grail of multiple sclerosis therapy Neurology 2010 74 1018 1019 10.1212/WNL.0b013e3181d6b165 20200341

[B33] Holick MF Vitamin D: a millenium perspective J Cell Biochem 2003 88 296 307 10.1002/jcb.10338 12520530

[B34] Ascherio A Munger KL Simon KC Vitamin D and multiple sclerosis Lancet Neurol 2010 9 599 612 10.1016/S1474-4422(10)70086-7 20494325

[B35] Holick MF Vitamin D deficiency N Engl J Med 2007 357 266 281 10.1056/NEJMra070553 17634462

[B36] Hagenau T Vest R Gissel TN Poulsen CS Erlandsen M Mosekilde L Vestergaard P Global vitamin D levels in relation to age, gender, skin pigmentation and latitude: an ecologic meta-regression analysis Osteoporos Int 2009 20 133 140 10.1007/s00198-008-0626-y 18458986

[B37] Database of the German Institut für Ernährungsinformation, Freudenstadt, Germany[http://www.ernaehrung.de/lebensmittel]

[B38] White JH Regulation of intracrine production of 1,25-dihydroxyvitamin D and its role in innate immune defense against infection Arch Biochem Biophys 2012 523 58 63 10.1016/j.abb.2011.11.006 22107948

[B39] Haussler MR Jurutka PW Mizwicki M Norman AW Vitamin D receptor (VDR)-mediated actions of 1α, 25(OH)2 vitamin D3: genomic and non-genomic mechanisms Best Pract Res Clin Endocrinol Metab 2011 25 543 559 10.1016/j.beem.2011.05.010 21872797

[B40] Carlberg C Molnar F Current status of vitamin D signaling and its therapeutic applications Curr Top Med Chem 2012 12 528 547 22242854 10.2174/156802612799436623

[B41] Holick MF Vitamin D status: measurement, interpretation, and clinical application Ann Epidemiol 2009 19 73 78 10.1016/j.annepidem.2007.12.001 18329892 PMC2665033

[B42] Souberbielle JC Body JJ Lappe JM Plebani M Shoenfeld Y Wang TJ Bischoff-Ferrari HA Cavalier E Ebeling PR Fardellone P Gandini S Gruson D Guérin AP Heickendorff L Hollis BW Ish-Shalom S Jean G Von Landenberg P Largura A Olsson T Pierrot-Deseilligny C Pilz S Tincani A Valcour A Zittermann A Vitamin D and musculoskeletal health, cardiovascular disease, autoimmunity and cancer: Recommendations for clinical practice Autoimmun Rev 2010 9 709 715 10.1016/j.autrev.2010.06.009 20601202

[B43] Bischoff-Ferrari HA Optimal serum 25-hydroxyvitamin D levels for multiple health outcomes Adv Exp Med Biol 2008 624 55 71 10.1007/978-0-387-77574-6_5 18348447

[B44] Thomas MK Lloyd-Jones DM Thadhani RI Shaw AC Deraska DJ Kitch BT Vamvakas EC Dick IM Prince RL Finkelstein JS Hypovitaminosis D in medical inpatients N Engl J Med 1998 338 777 783 10.1056/NEJM199803193381201 9504937

[B45] Holick MF High prevalence of vitamin D inadequacy and implications for health Mayo Clin Proc 2006 81 353 373 10.4065/81.3.353 16529140

[B46] Hintzpeter B Mensink GBM Thierfelder W Müller MJ Scheidt-Nave C Vitamin D status and health correlates among German adults Eur J Clin Nutr 2008 62 1079 1089 10.1038/sj.ejcn.1602825 17538533

[B47] Peelen E Knippenberg S Muris AH Thewissen M Smolders J Tervaert JWC Hupperts R Damoiseaux J Effects of vitamin D on the peripheral adaptive immune system: a review Autoimmun Rev 2011 10 733 743 10.1016/j.autrev.2011.05.002 21621002

[B48] Fleet JC DeSmet M Johnson R Li Y Vitamin D and cancer: a review of molecular mechanisms Biochem J 2012 441 61 76 10.1042/BJ20110744 22168439 PMC4572477

[B49] Grant WB An estimate of the global reduction in mortality rates through doubling vitamin D levels Eur J Clin Nutr 2011 65 1016 1026 10.1038/ejcn.2011.68 21731036

[B50] Deluca HF Cantorna MT Vitamin D: its role and uses in immunology FASEB J 2001 15 2579 2585 10.1096/fj.01-0433rev 11726533

[B51] Correale J Ysrraelit MC Gaitán MI Immunomodulatory effects of Vitamin D in multiple sclerosis Brain 2009 132 1146 1160 10.1093/brain/awp033 19321461

[B52] Di Rosa M Malaguarnera M Nicoletti F Malaguarnera L Vitamin D3: a helpful immuno-modulator Immunology 2011 134 123 139 10.1111/j.1365-2567.2011.03482.x 21896008 PMC3194221

[B53] Almerighi C Sinistro A Cavazza A Ciaprini C Rocchi G Bergamini A 1Alpha,25-dihydroxyvitamin D3 inhibits CD40L-induced pro-inflammatory and immunomodulatory activity in human monocytes Cytokine 2009 45 190 197 10.1016/j.cyto.2008.12.009 19186073

[B54] Piemonti L Monti P Sironi M Fraticelli P Leone BE Dal Cin E Allavena P Di Carlo V Vitamin D3 affects differentiation, maturation, and function of human monocyte-derived dendritic cells J Immunol 2000 164 4443 4451 10779743 10.4049/jimmunol.164.9.4443

[B55] Griffin MD Lutz W Phan VA Bachman LA McKean DJ Kumar R Dendritic cell modulation by 1alpha,25 dihydroxyvitamin D3 and its analogs: a vitamin D receptor-dependent pathway that promotes a persistent state of immaturity in vitro and in vivo Proc Natl Acad Sci USA 2001 98 6800 6805 10.1073/pnas.121172198 11371626 PMC34433

[B56] Lemire JM Adams JS Sakai R Jordan SC 1 alpha,25-dihydroxyvitamin D3 suppresses proliferation and immunoglobulin production by normal human peripheral blood mononuclear cells J Clin Invest 1984 74 657 661 10.1172/JCI111465 6611355 PMC370520

[B57] Chen S Sims GP Chen XX Gu YY Chen S Lipsky PE Modulatory effects of 1,25-dihydroxyvitamin D3 on human B cell differentiation J Immunol 2007 179 1634 1647 17641030 10.4049/jimmunol.179.3.1634

[B58] Bhalla AK Amento EP Serog B Glimcher LH 1,25-dihydroxyvitamin D3 inhibits antigen-induced T cell activation J Immunol 1984 133 1748 1754 6206136

[B59] Boonstra A Barrat FJ Crain C Heath VL Savelkoul HF O'Garra A 1alpha,25-Dihydroxyvitamin d3 has a direct effect on naive CD4(+) T cells to enhance the development of Th2 cells J Immunol 2001 167 4974 4980 11673504 10.4049/jimmunol.167.9.4974

[B60] Yu S Cantorna MT Epigenetic reduction in invariant NKT cells following in utero vitamin D deficiency in mice J Immunol 2011 186 1384 1390 10.4049/jimmunol.1002545 21191070 PMC3127168

[B61] Smolders J Thewissen M Peelen E Menheere P Tervaert JWC Damoiseaux J Hupperts R Vitamin D status is positively correlated with regulatory T cell function in patients with multiple sclerosis PLoS One 2009 4 e6635 10.1371/journal.pone.0006635 19675671 PMC2721656

[B62] Döring A Paul F Dörr J Vitamin D and multiple sclerosis: the role for risk of disease and treatment Nervenarzt 2012 10.1007/s00115-012-3645-z 23052893

[B63] Eyles DW Smith S Kinobe R Hewison M McGrath JJ Distribution of the vitamin D receptor and 1 alpha-hydroxylase in human brain J Chem Neuroanat 2005 29 21 30 10.1016/j.jchemneu.2004.08.006 15589699

[B64] Garcion E Wion-Barbot N Montero-Menei CN Berger F Wion D New clues about vitamin D functions in the nervous system Trends Endocrinol Metab 2002 13 100 105 10.1016/S1043-2760(01)00547-1 11893522

[B65] de Abreu DAF Eyles D Féron F Vitamin D, a neuro-immunomodulator: implications for neurodegenerative and autoimmune diseases Psychoneuroendocrinology 2009 34 Suppl 1 S265 S277 19545951 10.1016/j.psyneuen.2009.05.023

[B66] Dyment DA Sadovnick AD Ebers GC Sadnovich AD Genetics of multiple sclerosis Hum Mol Genet 1997 6 1693 1698 10.1093/hmg/6.10.1693 9300661

[B67] Sadovnick AD Genetic background of multiple sclerosis Autoimmun Rev 2012 11 163 166 10.1016/j.autrev.2011.05.007 21619948

[B68] Ramagopalan SV Maugeri NJ Handunnetthi L Lincoln MR Orton S-M Dyment DA Deluca GC Herrera BM Chao MJ Sadovnick AD Ebers GC Knight JC Expression of the multiple sclerosis-associated MHC class II Allele HLA-DRB1*1501 is regulated by vitamin D PLoS Genet 2009 5 e1000369 10.1371/journal.pgen.1000369 19197344 PMC2627899

[B69] Ramagopalan SV Dyment DA Cader MZ Morrison KM Disanto G Morahan JM Berlanga-Taylor AJ Handel A De Luca GC Sadovnick AD Lepage P Montpetit A Ebers GC Rare variants in the CYP27B1 gene are associated with multiple sclerosis Ann Neurol 2011 70 881 886 10.1002/ana.22678 22190362

[B70] Torkildsen Ø Knappskog PM Nyland HI Myhr KM Vitamin D-dependent rickets as a possible risk factor for multiple sclerosis Arch Neurol 2008 65 809 811 10.1001/archneur.65.6.809 18541802

[B71] Ramagopalan SV Hanwell HEC Giovannoni G Knappskog PM Nyland HI Myhr K-M Ebers GC Torkildsen O Vitamin D-dependent rickets, HLA-DRB1, and the risk of multiple sclerosis Arch Neurol 2010 67 1034 1035 10.1001/archneurol.2010.182 20697062

[B72] Lemire JM Archer DC 1,25-dihydroxyvitamin D3 prevents the in vivo induction of murine experimental autoimmune encephalomyelitis J Clin Invest 1991 87 1103 1107 10.1172/JCI115072 1705564 PMC329907

[B73] Cantorna MT Hayes CE DeLuca HF 1,25-Dihydroxyvitamin D3 reversibly blocks the progression of relapsing encephalomyelitis, a model of multiple sclerosis Proc Natl Acad Sci USA 1996 93 7861 7864 10.1073/pnas.93.15.7861 8755567 PMC38839

[B74] Pedersen LB Nashold FE Spach KM Hayes CE 1,25-dihydroxyvitamin D3 reverses experimental autoimmune encephalomyelitis by inhibiting chemokine synthesis and monocyte trafficking J Neurosci Res 2007 85 2480 2490 10.1002/jnr.21382 17600374

[B75] Spach KM Hayes CE Vitamin D3 confers protection from autoimmune encephalomyelitis only in female mice J Immunol 2005 175 4119 4126 16148162 10.4049/jimmunol.175.6.4119

[B76] Becklund BR Severson KS Vang SV DeLuca HF UV radiation suppresses experimental autoimmune encephalomyelitis independent of vitamin D production Proc Natl Acad Sci USA 2010 107 6418 6423 10.1073/pnas.1001119107 20308557 PMC2851981

[B77] Wergeland S Torkildsen O Myhr KM Aksnes L Mørk SJ Bø L Dietary vitamin D3 supplements reduce demyelination in the cuprizone model PLoS One 2011 6 e26262 10.1371/journal.pone.0026262 22028844 PMC3197632

[B78] Acheson ED Bachrach CA Wright FM Some comments on the relationship of the distribution of multiple sclerosis to latitude, solar radiation, and other variables Acta Psychiatr Scand Suppl 1960 35 132 147 10.1111/j.1600-0447.1960.tb08674.x 13681205

[B79] Kurtzke JF An evaluation of the geographic distribution of multiple sclerosis Acta Neurol Scand 1966 42 Suppl 19 91 5929916 10.1111/j.1600-0404.1966.tb02008.x

[B80] Swank RL Lerstad O Strom A Backer J Multiple sclerosis in rural Norway its geographic and occupational incidence in relation to nutrition N Engl J Med 1952 246 722 728 14929306 10.1056/NEJM195205082461901

[B81] Kurtzke JF Beebe GW Norman JE Jr Epidemiology of multiple sclerosis in US veterans: III. Migration and the risk of MS Neurology 1985 35 672 678 10.1212/WNL.35.5.672 3873023

[B82] Willer CJ Dyment DA Sadovnick AD Rothwell PM Murray TJ Ebers GC Timing of birth and risk of multiple sclerosis: population based study BMJ 2005 330 120 10.1136/bmj.38301.686030.63 15585537 PMC544426

[B83] Sotgiu S Pugliatti M Sotgiu MA Fois ML Arru G Sanna A Rosati G Seasonal fluctuation of multiple sclerosis births in Sardinia J Neurol 2006 253 38 44 10.1007/s00415-005-0917-6 16021348

[B84] Salzer J Svenningsson A Sundström P Season of birth and multiple sclerosis in Sweden Acta Neurol Scand 2010 122 70 73 20597868 10.1111/j.1600-0404.2010.01396.x

[B85] Dobson R Giovannoni G Ramagopalan S The month of birth effect in multiple sclerosis: systematic review, meta-analysis and effect of latitude J Neurol Neurosurg Psychiatr 2012 10.1136/jnnp-2012-303934 23152637

[B86] Van der Mei IAF Ponsonby AL Dwyer T Blizzard L Simmons R Taylor BV Butzkueven H Kilpatrick T Past exposure to sun, skin phenotype, and risk of multiple sclerosis: case–control study BMJ 2003 327 316 10.1136/bmj.327.7410.316 12907484 PMC169645

[B87] Islam T Gauderman WJ Cozen W Mack TM Childhood sun exposure influences risk of multiple sclerosis in monozygotic twins Neurology 2007 69 381 388 10.1212/01.wnl.0000268266.50850.48 17646631

[B88] Dalmay F Bhalla D Nicoletti A Cabrera-Gomez JA Cabre P Ruiz F Druet-Cabanac M Dumas M Preux PM Multiple sclerosis and solar exposure before the age of 15 years: case–control study in Cuba, Martinique and Sicily Mult Scler 2010 16 899 908 10.1177/1352458510366856 20463038

[B89] Kampman MT Wilsgaard T Mellgren SI Outdoor activities and diet in childhood and adolescence relate to MS risk above the Arctic Circle J Neurol 2007 254 471 477 10.1007/s00415-006-0395-5 17377831

[B90] McDowell TY Amr S Culpepper WJ Langenberg P Royal W Bever C Bradham DD Sun exposure, vitamin D and age at disease onset in relapsing multiple sclerosis Neuroepidemiology 2011 36 39 45 10.1159/000322512 21160231

[B91] McDowell TY Amr S Culpepper WJ Langenberg P Royal W Bever C Bradham DD Sun exposure, vitamin D intake and progression to disability among veterans with progressive multiple sclerosis Neuroepidemiology 2011 37 52 57 10.1159/000329258 21822026

[B92] Van der Mei IAF Blizzard L Ponsonby AL Dwyer T Validity and reliability of adult recall of past sun exposure in a case–control study of multiple sclerosis Cancer Epidemiol Biomarkers Prev 2006 15 1538 1544 10.1158/1055-9965.EPI-05-0969 16896046

[B93] Lucas RM Ponsonby AL Dear K Valery PC Pender MP Taylor BV Kilpatrick TJ Dwyer T Coulthard A Chapman C Van der Mei I Williams D McMichael AJ Sun exposure and vitamin D are independent risk factors for CNS demyelination Neurology 2011 76 540 548 10.1212/WNL.0b013e31820af93d 21300969

[B94] Hart PH Gorman S Finlay-Jones JJ Modulation of the immune system by UV radiation: more than just the effects of vitamin D? Nat Rev Immunol 2011 11 584 596 10.1038/nri3045 21852793

[B95] Jin Y De Pedro-Cuesta J Söderström M Stawiarz L Link H Seasonal patterns in optic neuritis and multiple sclerosis: a meta-analysis J Neurol Sci 2000 181 56 64 10.1016/S0022-510X(00)00408-1 11099713

[B96] Handel AE Disanto G Jarvis L McLaughlin R Fries A Ebers GC Ramagopalan SV Seasonality of admissions with multiple sclerosis in Scotland Eur J Neurol 2011 18 1109 1111 10.1111/j.1468-1331.2010.03318.x 21749578

[B97] Meier DS Balashov KE Healy B Weiner HL Guttmann CRG Seasonal prevalence of MS disease activity Neurology 2010 75 799 806 10.1212/WNL.0b013e3181f0734c 20805526 PMC2938966

[B98] Auer DP Schumann EM Kümpfel T Gössl C Trenkwalder C Seasonal fluctuations of gadolinium-enhancing magnetic resonance imaging lesions in multiple sclerosis Ann Neurol 2000 47 276 277 10.1002/1531-8249(200002)47:2<276::AID-ANA28>3.0.CO;2-1 10665507

[B99] Tremlett H Van der Mei IAF Pittas F Blizzard L Paley G Mesaros D Woodbaker R Nunez M Dwyer T Taylor BV Ponsonby AL Monthly ambient sunlight, infections and relapse rates in multiple sclerosis Neuroepidemiology 2008 31 271 279 18971584 10.1159/000166602

[B100] Mirzaei F Michels KB Munger K O'Reilly E Chitnis T Forman MR Giovannucci E Rosner B Ascherio A Gestational vitamin D and the risk of multiple sclerosis in offspring Ann Neurol 2011 70 30 40 10.1002/ana.22456 21786297 PMC3205990

[B101] Banwell B Bar-Or A Arnold DL Sadovnick D Narayanan S McGowan M O'Mahony J Magalhaes S Hanwell H Vieth R Tellier R Vincent T Disanto G Ebers G Wambera K Connolly MB Yager J Mah JK Booth F Sebire G Callen D Meaney B Dilenge ME Lortie A Pohl D Doja A Venketaswaran S Levin S Macdonald EA Meek D Clinical, environmental, and genetic determinants of multiple sclerosis in children with acute demyelination: a prospective national cohort study Lancet Neurol 2011 10 436 445 10.1016/S1474-4422(11)70045-X 21459044

[B102] Munger KL Levin LI Hollis BW Howard NS Ascherio A Serum 25-hydroxyvitamin D levels and risk of multiple sclerosis JAMA 2006 296 2832 2838 10.1001/jama.296.23.2832 17179460

[B103] Munger KL Zhang SM O'Reilly E Hernán MA Olek MJ Willett WC Ascherio A Vitamin D intake and incidence of multiple sclerosis Neurology 2004 62 60 65 10.1212/01.WNL.0000101723.79681.38 14718698

[B104] Soilu-Hänninen M Airas L Mononen I Heikkilä A Viljanen M Hänninen A 25-Hydroxyvitamin D levels in serum at the onset of multiple sclerosis Mult Scler 2005 11 266 271 10.1191/1352458505ms1157oa 15957505

[B105] Simpson S Jr Taylor B Blizzard L Ponsonby AL Pittas F Tremlett H Dwyer T Gies P Van der Mei I Higher 25-hydroxyvitamin D is associated with lower relapse risk in multiple sclerosis Ann Neurol 2010 68 193 203 20695012 10.1002/ana.22043

[B106] Pierrot-Deseilligny C Rivaud-Péchoux S Clerson P De Paz R Souberbielle JC Relationship between 25-OH-D serum level and relapse rate in multiple sclerosis patients before and after vitamin D supplementation Ther Adv Neurol Disord 2012 5 187 198 10.1177/1756285612447090 22783368 PMC3388527

[B107] Runia TF Hop WCJ De Rijke YB Buljevac D Hintzen RQ Lower serum vitamin D levels are associated with a higher relapse risk in multiple sclerosis Neurology 2012 79 261 266 10.1212/WNL.0b013e31825fdec7 22700811

[B108] Mowry EM Krupp LB Milazzo M Chabas D Strober JB Belman AL McDonald JC Oksenberg JR Bacchetti P Waubant E Vitamin D status is associated with relapse rate in pediatric-onset multiple sclerosis Ann Neurol 2010 67 618 624 20437559 10.1002/ana.21972

[B109] Mowry EM Waubant E McCulloch CE Okuda DT Evangelista AA Lincoln RR Gourraud P-A Brenneman D Owen MC Qualley P Bucci M Hauser SL Pelletier D Vitamin D status predicts new brain magnetic resonance imaging activity in multiple sclerosis Ann Neurol 2012 72 234 240 10.1002/ana.23591 22926855 PMC3430977

[B110] Løken-Amsrud KI Holmøy T Bakke SJ Beiske AG Bjerve KS Bjørnarå BT Hovdal H Lilleås F Midgard R Pedersen T Benth JS Sandvik L Torkildsen O Wergeland S Myhr K-M Vitamin D and disease activity in multiple sclerosis before and during interferon-β treatment Neurology 2012 79 267 273 10.1212/WNL.0b013e31825fdf01 22700809

[B111] Holmøy T Moen SM Gundersen TA Holick MF Fainardi E Castellazzi M Casetta I 25-hydroxyvitamin D in cerebrospinal fluid during relapse and remission of multiple sclerosis Mult Scler 2009 15 1280 1285 10.1177/1352458509107008 19808741

[B112] Goldberg P Fleming MC Picard EH Multiple sclerosis: decreased relapse rate through dietary supplementation with calcium, magnesium and vitamin D Med Hypotheses 1986 21 193 200 10.1016/0306-9877(86)90010-1 3537648

[B113] Kimball SM Ursell MR O'Connor P Vieth R Safety of vitamin D3 in adults with multiple sclerosis Am J Clin Nutr 2007 86 645 651 17823429 10.1093/ajcn/86.3.645

[B114] Burton JM Kimball S Vieth R Bar-Or A Dosch H-M Cheung R Gagne D D'Souza C Ursell M O'Connor P A phase I/II dose-escalation trial of vitamin D3 and calcium in multiple sclerosis Neurology 2010 74 1852 1859 10.1212/WNL.0b013e3181e1cec2 20427749 PMC2882221

[B115] Mahon BD Gordon SA Cruz J Cosman F Cantorna MT Cytokine profile in patients with multiple sclerosis following vitamin D supplementation J Neuroimmunol 2003 134 128 132 10.1016/S0165-5728(02)00396-X 12507780

[B116] Stein MS Liu Y Gray OM Baker JE Kolbe SC Ditchfield MR Egan GF Mitchell PJ Harrison LC Butzkueven H Kilpatrick TJ A randomized trial of high-dose vitamin D2 in relapsing-remitting multiple sclerosis Neurology 2011 77 1611 1618 10.1212/WNL.0b013e3182343274 22025459

[B117] Grimaldi L Barkhof F Beelke M Burton J Holmoy T Hupperts R Killestein J Rieckmann P Schluep M Smolders J A randomized trial of high-dose vitamin D2 in relapsing-remitting multiple sclerosis Neurology 2012 78 841 22411961 10.1212/01.wnl.0000413180.13413.ce

[B118] Shaygannejad V Janghorbani M Ashtari F Dehghan H Effects of adjunct low-dose vitamin d on relapsing-remitting multiple sclerosis progression: preliminary findings of a randomized placebo-controlled trial Mult Scler Int 2012 2012 452541 22567287 10.1155/2012/452541 PMC3337486

[B119] Soilu-Hänninen M Aivo J Lindström B-M Elovaara I Sumelahti M-L Färkkilä M Tienari P Atula S Sarasoja T Herrala L Keskinarkaus I Kruger J Kallio T Rocca MA Filippi M A randomised, double blind, placebo controlled trial with vitamin D3 as an add on treatment to interferon β-1b in patients with multiple sclerosis J Neurol Neurosurg Psychiatry 2012 83 565 571 10.1136/jnnp-2011-301876 22362918

[B120] Aivo J Lindsröm B-M Soilu-Hänninen M A randomised, double-blind, placebo-controlled trial with vitamin D3 in MS: subgroup analysis of patients with baseline disease activity despite interferon treatment Mult Scler Int 2012 2012 802796 22919492 10.1155/2012/802796 PMC3420140

[B121] Kampman MT Steffensen LH Mellgren SI Jørgensen L Effect of vitamin D3 supplementation on relapses, disease progression and measures of function in persons with multiple sclerosis: exploratory outcomes from a double-blind randomised controlled trial Mult Scler 2012 18 1144 1151 10.1177/1352458511434607 22354743

[B122] Smolders J Hupperts R Barkhof F Grimaldi LME Holmoy T Killestein J Rieckmann P Schluep M Vieth R Hostalek U Ghazi-Visser L Beelke M Efficacy of vitamin D(3) as add-on therapy in patients with relapsing-remitting multiple sclerosis receiving subcutaneous interferon beta-1a: a Phase II, multicenter, double-blind, randomized, placebo-controlled trial J Neurol Sci 2011 311 44 49 10.1016/j.jns.2011.04.013 21620416

[B123] Dörr J Ohlraun S Skarabis H Paul F Efficacy of Vitamin D Supplementation in Multiple Sclerosis (EVIDIMS Trial): study protocol for a randomized controlled trial Trials 2012 13 15 10.1186/1745-6215-13-15 22316314 PMC3298796

[B124] Marcus JF Shalev SM Harris CA Goodin DS Josephson SA Severe hypercalcemia following vitamin D supplementation in a patient with multiple sclerosis: a note of caution Arch Neurol 2012 69 129 132 10.1001/archneurol.2011.1199 22232355

[B125] Wingerchuk DM Lesaux J Rice GPA Kremenchutzky M Ebers GC A pilot study of oral calcitriol (1,25-dihydroxyvitamin D3) for relapsing-remitting multiple sclerosis J Neurol Neurosurg Psychiatr 2005 76 1294 1296 10.1136/jnnp.2004.056499 16107372 PMC1739797

[B126] Jagannath VA Fedorowicz Z Asokan GV Robak EW Whamond L Vitamin D for the management of multiple sclerosis Cochrane Database Syst Rev 2010 12 CD008422 21154396 10.1002/14651858.CD008422.pub2

[B127] Hathcock JN Shao A Vieth R Heaney R Risk assessment for vitamin D Am J Clin Nutr 2007 85 6 18 17209171 10.1093/ajcn/85.1.6

[B128] Golubnitschaja O Costigliola V EPMA General report & recommendations in predictive, preventive and personalised medicine 2012: white paper of the European Association for Predictive, Preventive and Personalised Medicine EPMA J 2012 3 14 10.1186/1878-5085-3-14 23116135 PMC3485619

